# Dataset on target chemical and bioassay analysis—Exploring contaminants of emerging concern in a low mountain river of central Germany

**DOI:** 10.1016/j.dib.2024.110510

**Published:** 2024-05-11

**Authors:** Fabian G. Weichert, Werner Brack, Mario Brauns, Patrick Fink, Sarah Johann, Martin Krauss, Henner Hollert

**Affiliations:** a Department Evolutionary Ecology & Environmental Toxicology, Faculty of Biological Sciences – Goethe University Frankfurt, Frankfurt am Main, Germany; b Department of Exposure Science, Helmholtz Centre for Environmental Research – UFZ, Leipzig, Germany; c Department of River Ecology, Helmholtz Centre for Environmental Research – UFZ, Magdeburg, Germany; d Department of Aquatic Ecosystem Analysis and Management, Helmholtz Centre for Environmental Research – UFZ, Magdeburg, Germany; e LOEWE Centre for Translational Biodiversity Genomics (LOEWE-TBG), Frankfurt am Main, Germany; f Department Environmental Media Related Ecotoxicology, Fraunhofer Institute for Molecular Biology and Applied Ecology IME, Frankfurt am Main, Germany

**Keywords:** Chemical pollution, Chemical screening, Bioanalytical screening, Effect-based methods

## Abstract

Chemical pollution of the aquatic environment is nowadays characterised by increasing levels of anthropogenic organic compounds at low concentrations and is recognised as one of the main drivers of the deteriorated ecological state of European waterbodies. To improve the understanding of the impact of chemical pollution in surface waters, a combined approach of chemical and bioanalytical testing is considered necessary for effective ecologically oriented water management. For this dataset, six 25-L water samples were collected at six sampling sites along the Holtemme River in Central Germany using large-volume solid phase extraction. All samples were analysed by targeted high-resolution liquid chromatography–mass spectrometry (LC–MS) and a selected bioanalytical test battery using effect-based methods. These methods included cytotoxicity assessment, several mechanism-specific CALUX^Ⓡ^ tests to identify endocrine and oxidative stress-related effects and the fish embryo acute toxicity test to investigate (sub)lethal effects in the model species *Danio rerio*. This approach provided a dataset that offers a longitudinal characterisation of the chemical pollution and ecotoxicological impacts. The combination of chemical analysis and effect-based analysis is valuable for future studies as it will help researchers, risk assessors and authorities to identify hot spots of chemical pollution, monitor environmental quality standards and recommend mitigation strategies.

Specifications Table

**Table:** 

Subject	Environmental Science / Environmental Chemistry / Pollution / Hydrology and Water quality
Specific subject area	Ecotoxicology, specifically environmental risk assessment
Type of data	Tables Raw Processed
Data collection	Samples were taken by large-volume solid phase extraction of 25 L of river water using cartridges with the sorbent CHROMABOND HR-X (Macherey-Nagel). The cartridges were freeze-dried and eluted with ethyl acetate, methanol, methanol containing formic acid and methanol containing ammonia. The extracts were evaporated (Multivapor P-6/Rotavapor R-300, BÜCHI Labortechnik AG) before they were subjected to chemical analysis and bioanalytic assessment. The chemical data were obtained by means of liquid chromatography using a UltiMate 3000 LC system (Thermo Fisher Scientific Inc., Waltham, USA), and high-resolution mass spectrometry equipped with electrospray ionisation (ESI), using an Orbitrap MS (Q Exactive Plus, Thermo Fisher Scientific). After peak detection in MZmine 2.38, blank correction, calibration, and quantification were performed in the R package {MZquant}. The target list included compounds from various chemical groups such as pesticides, biocides, personal care products, pharmaceuticals, industrials and natural compounds. The method detection limits were calculated as outlined by US EPA methodologies. The cytotoxicity of each sample was analysed by means of the neutral red retention assay using a Tecan Spark^Ⓡ^ multimode reader (Tecan Trading AG, Männedorf, Switzerland) to quantify the fluorescence of neutral red. Bioanalytical data were obtained by means of the CALUX^Ⓡ^ (Chemically Activated Luciferase gene eXpression) test system (BioDetection Systems BV, Amsterdam, The Netherlands), using a Tecan Spark^Ⓡ^ multimode reader (Tecan Trading AG, Männedorf, Switzerland) to quantify luminescence. Calculations of bioanalytical equivalent values as well as limits of quantification were performed in Excel (Microsoft Corporation, Redmond, Washington, USA), using macro-enabled templates provided by BioDetection Systems BV. Data for fish acute toxicity test with wild-type zebrafish (*Danio rerio*) were obtained following the OECD Guideline 236 using eggs from an in-house breeding facility.
Data source location	The data are stored at the Department Evolutionary Ecology & Environmental Toxicology, Faculty of Biological Sciences – Goethe University Frankfurt, Frankfurt am Main, Germany and the Department of Exposure Science, Helmholtz Centre for Environmental Research – UFZ, Leipzig, Germany The data were collected from **H1** (51°49′01.1″N, 10°43′26.6″E), **H2** (51°50′59.4″N, 10°47′49.4″E), **H3** (51°52′04.4″N, 10°52′24.8″E), **H4** (51°53′06.1″N, 10°57′47.1″E), **H5** (51°54′15.6″N, 11°03′42.5″E) and **H6** (51°54′19.6″N, 11°04′02.8″E).
Data accessibility	Repository name: Zenodo Data identification number: 10.5281/zenodo.10892039 [1] Direct URL to data: https://zenodo.org/records/10892039

## 1 Value of the Data

• The presented dataset offers valuable insight into the state of contamination along a river gradient in Central Germany flowing through forested, urban as well as agricultural areas, as it provides a comprehensive overview of compounds of emerging concern, covering pesticides, pharmaceuticals, personal care products and industrial chemicals.
• The characterisation of the effect profile using bioanalytical tools adds valuable information to the ecotoxicological impact of surface water contamination.
• The combination of target chemical data and effect-based data is of value for future studies as it will help researchers, risk assessors and authorities to identify hot spots of chemical pollution, monitor environmental quality standards and recommend mitigation strategies.

## 2 Background

Water pollution in Europe is characterised by an ever-increasing number of anthropogenic organic compounds that are detected at low concentrations. Besides indirect inputs from agriculture, industry and urban surfaces, direct sources like conventional wastewater treatment plants without advanced treatment techniques, hospital effluents and industrial discharges are considered the main pathways for pollutants to enter the aquatic environment [2,3]. The consequences of this anthropogenic environmental pollution for biodiversity, ecosystems and their living constituents remain only inadequately comprehended and are often difficult or impossible to assess [4,5]. Nevertheless, chemical pollution is considered one of the main drivers of the deteriorated ecological state of European waterbodies [6] and combining the application of chemical analysis and effect-based (i.e. bioanalytical assays) tools is regarded as an important concept for ecology-orientated water management [2,7,8]. Here, we present a dataset comprising comprehensive chemical analysis and ecotoxicological effects characterisation in river water samples along a gradient of increasing anthropogenic influence. This dataset can be used to evaluate the state of pollution with organic compounds from anthropogenic sources along the river course using e.g., mixture risk assessment approaches, which could in parts be validated or contradicted with the bioanalytical data. Thus, the dataset can help to identify potential hot spots of pollution and pinpoint possible compounds of biological concern.

## 3 Data Description

**Fig. 1:**
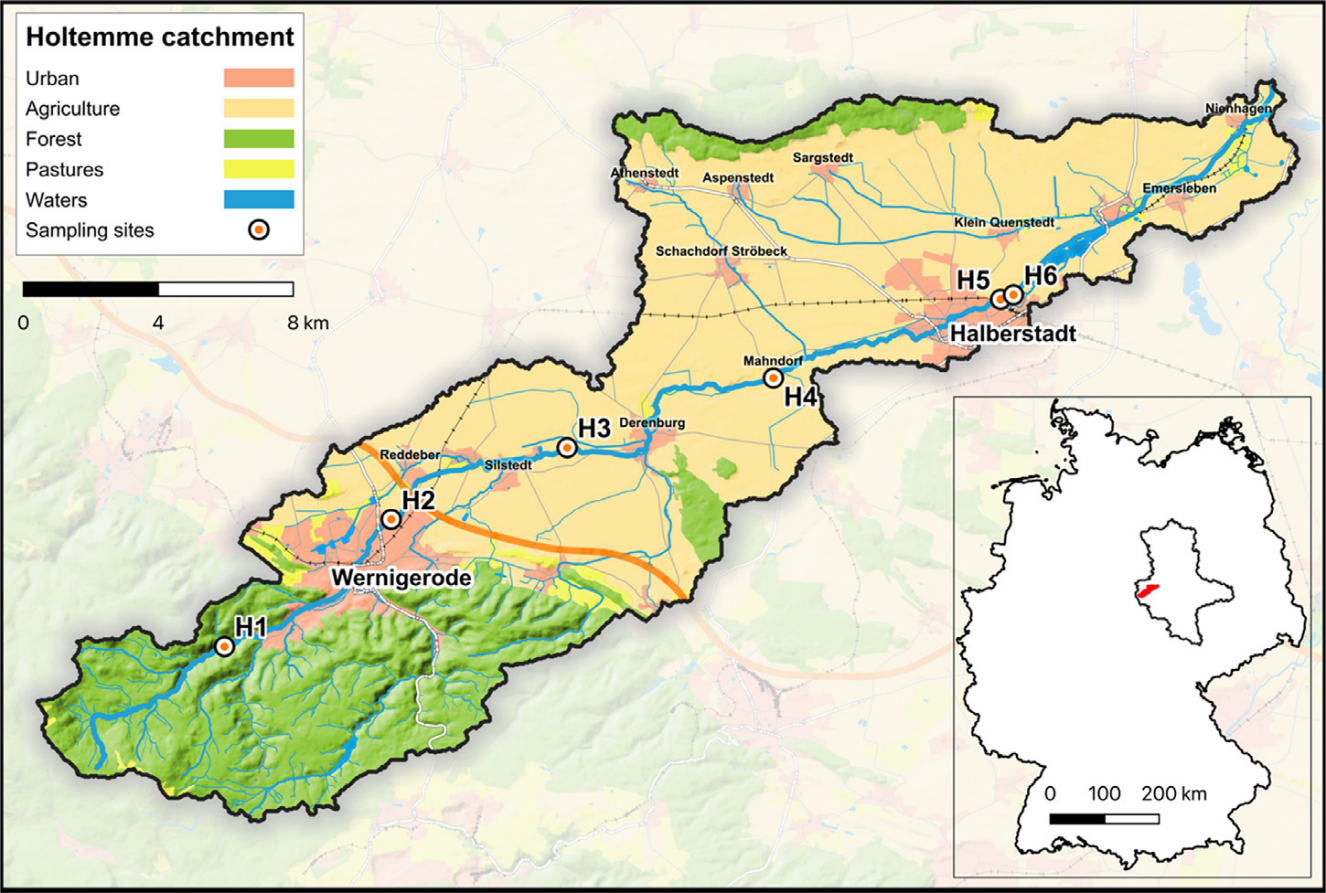
Representation of the Holtemme River catchment, showing the sampling sites and land-use within the area. Generated using European Union's Copernicus Land Monitoring Service information; https://doi.org/10.2909/71c95a07-e296-44fc-b22b-415f42acfdf0.

The dataset presented includes chemical and bioanalytical data for river water samples collected from six individual sampling sites along the Holtemme River catchment (Fig. 1) in Central Germany, using on-site Large-Volume Solid Phase Extraction (LVSPE).

The main dataset comprises five delimiter-separated values files using semicolon as delimiter (csv-format) and one Excel spreadsheet (xlsx-format) that can be downloaded from [1]. The file “LVSPE.csv” contains all chemical data, the file “EBM.csv” includes the processed data from effect-based methods (EBMs) and the files “FET.csv” and “NR.csv” contain the pre-processed data from the fish embryo toxicity test (FET) and neutral red retention assay (NR), respectively. Additionally, we provide the file “Water_parameters.csv”, which contains a set of water parameters determined during the sampling as well as the discharge at two gauges along the river during sampling (at sampling sites H1 and H4). Table 1 summarises the content of all reported columns in the csv-files. The Excel spreadsheet file “EBM_raw_data.xlsx” comprises eight individual sheets containing raw fluorescence and luminescence reads for the NR and all Chemically Activated Luciferase gene eXpression (CALUX^Ⓡ^) assays along with the respective pipetting scheme. More details are summarised in Table 2.

**Table 1:** Description of the columns included in the presented dataset.

File name	Column names	Description
LVSPE.csv	Compound name	Common name of the compound measured
	MDL	Method detection limit in ng/L
	H1-H6	Concentrations of compounds in ng/L in samples from the sites H1 through H6
	Trip blank	Concentration of compounds in ng/L in the trip blank sample
	InChIKey	International Chemical Identifier
	SMILES	Simplified Molecular-Input Line-Entry System
	DTXSID	CompTox Chemicals Dashboard Identifier
	Compound class level 1–3	In-house class definition of measured compound in three different resolutions
NR.csv	Sample	Name of the sample
	Concentration / REF	Tested concentration in relative enrichment factor (REF)
	Cytotoxicity / %	Percentage of cytotoxicity normalised to negative control
EBM.csv	Effect-based method	The effect-based method used
	Endpoint	The endpoint of assay used; these can be bioanalytical equivalents (e.g., 17β-oestradiol-equivalent in ng/L) as well as lethal, effect or inhibitory concentrations (LCs, ECs, ICs)
	LOQ	Limit of quantification, where applicable
	Replication	Number of replicates, where applicable
	H1-H6	Effects for the samples from the sites H1 through H6, using the same unit as defined in the endpoint column
	Trip blank	Effect for the trip blank sample, using the same unit as defined in the endpoint column
FET.csv	Sample	Name of the sample
	Concentration / REF	Tested concentration in relative enrichment factor (REF)
	Mortality / %	Percentage of lethal deformities scored
	Effects / %	Percentage of lethal and sublethal deformities scored
	Hatching / %	Percentage of hatched embryos
Water_parameters.csv	Sampling site	Code of the sampling site
	pH	pH at the sampling site during sampling
	Temperature / °C	Temperature at the sampling site during sampling
	Oxygen concentration / mg/L	Oxygen concentration at the sampling site during sampling
	Oxygen saturation / %	Respective oxygen concentration saturation
	Conductivity / µS/cm	Conductivity at the sampling site during sampling
	Discharge / m^3^/s	Discharge during sampling, the gauges were within 200 m of the sampling site

**Table 2:** Description of the Excel spreadsheet containing the raw fluorescence and luminescence reads for the respective bioassays along with pipetting scheme used.

File name	Sheet name	Description
EBM_raw_data.xlsx	NR_raw	Sheet containing raw fluorescence reads from the neutral red retention assay. Column A and B contain the plate number and the well row of each plate, respectively. The columns C to M contain the relative fluorescence reads, columns N to X the respective pipetting scheme.
	AR_raw Anti-AR_raw ER_raw Anti-ER_raw Nrf2_raw PR_raw GR_raw	The sheets AR_raw, anti-AR_raw, ER_raw, anti-ER_raw, Nrf2_raw, PR_raw and GR_raw contain the raw luminescence reads from the AR-, anti-AR-, ER-, anti-ER-, Nrf2-, PR- and GR-CALUX^Ⓡ^, respectively. Column A and B contain the plate number and the well row of each plate, respectively. The columns C to L contain the relative luminescence reads, columns M to V the respective pipetting scheme.

## 4 Experimental Design, Materials and Methods

### 4.1 Sampling sites, sampling and sample preparation

The Holtemme River is a low mountain stream located in Central Germany that springs in the Harz/Saxony-Anhalt Nature Park and flows into the Bode River as a left tributary after approx. 47 km. The Holtemme thus belongs to the Elbe/Saale/Bode river system and has a catchment area of ca. 282 km^2^. It represents a watercourse that has only been marginally altered by human influences in its upper reaches, but is increasingly subjected to hydromorphological changes and increasing anthropogenic stressors in its downstream reaches (Fig. 1). The variety of chemical pollution sources ranges from diffuse sources, e.g. from agricultural land or urban areas, to temporary point sources, such as rainwater retention basins, to continuous point sources, such as the two wastewater treatment plants Silstedt (80,000 population equivalents; commissioned: 1996) and Halberstadt (60,000 population equivalents; commissioned before 1990, last expansion: 2000) [9].

Six priority sampling sites along the river course (H1, H2, H3, H4, H5 and H6) were chosen to cover the various anthropogenic impact types. The reference site (H1) was located in the headwaters of the river and reflects negligible human influence on the waterbody and riparian zone. Within the City of Wernigerode, the sampling site H2 was selected as urban impact site. Positioned after the outlet of the Silstedt wastewater treatment plant (WWTP), H3 was characterising a site affected by WWTP effluent. Surrounded by arable land, H4 represented a site where impacts of agricultural land use were likely to occur. A second urban impact site (H5), located after the river passed the City of Halberstadt, and a second WWTP affected site (H6) after receiving effluent from the WWTP Halberstadt, were selected. The sampling sites H1 – H5 were between 6 and 8 km distanced from each other, while H6 was approximately 400 m downstream of H5 (Fig. 1).

In September 2021, 25 L of water were sampled over the course of three hours at each site, using LVSPE [10]. At each sampling location, 50 aliquots of 500 mL water were collected from the middle of the stream at a depth of 20–30 cm. The water was pumped to the LVSPE machine through a PTFE tubing (≤ 6 m) equipped with a custom-made stainless steel mesh filter (mesh size: 0.85 mm) to prevent solid matter from reaching the machine. In the machine, the water was pumped through a prefilter (Sartopure GF+ MidiCap, 0.65 μm pore size, Sartorius) into a borosilicate glass dosing system (volume: 500 mL) and subsequently through a conditioned (LC-MS grade ethyl acetate, methanol and water) custom-made cartridge, filled with 10 g of sorbent (CHROMABOND HR-X, Macherey-Nagel, Düren, Germany). For a trip blank control, an additional cartridge was carried during the entire sampling campaign at the same conditions as the sample cartridges.

Upon arrival in the laboratory, the cartridges were blown dry with nitrogen gas and subsequently freeze-dried before elution. Elution was done using 100 mL of ethyl acetate, 100 mL of methanol, 100 mL of methanol containing 1 % (v/v) formic acid and 100 mL of methanol with 2 % (v/v) 7 N ammonia. The extracts were evaporated (Multivapor P-6/Rotavapor R-300, BÜCHI Labortechnik AG, Flawil, Switzerland) to reach a final relative enrichment factor (REF) of 40,000, the solvent was exchanged to DMSO (LC-MS grade) and extracts were stored at −20 °C until further use.

### 4.2 Liquid chromatography-HRMS analysis

The extracts were reconstituted in LC-MS grade methanol at a REF 1000. For analysis, LVSPE samples or the trip blank sample (REF 1000) were spiked with an internal isotope-labelled standard mixture. Aliquots of spiked LVSPE samples and the trip blank were injected into an UltiMate 3000 LC system (Thermo Fisher Scientific), using a reversed-phase column for separation. Mass spectrometry data were obtained by electrospray ionisation (ESI) in positive and negative mode, using a Q Exactive Plus quadrupole Orbitrap (Thermo Fisher Scientific). Matrix-matched calibration was used. For calibration, pristine river water (Wormsgraben, upper Harz mountains, Saxony-Anhalt Nature Park) spiked with target compounds was extracted by laboratory-scale solid phase extraction method with the same sorbent and elution method as detailed above. Data evaluation was done as described in Finckh et al. [11]. Briefly, after peak detection in MZmine 2.38, the peak list was exported as csv-file and blank correction, calibration as well as internal standard quantification of target compounds was performed in the R package {MZquant} [12]. The respective method detection limits (MDLs) were calculated as outlined by the US EPA methodology [13], based on the standard deviation of replicated analysis of five matrix-matched calibration standards followed by a *t*-test.

### 4.3 Effect-based methods

#### 4.3.1 Neutral red retention assay

The neutral red retention (NR) assay was used to assess the cytotoxicity of the sample extracts to human osteosarcoma cell line U2-OS as described in Repetto et al. [14]. In triplicates, the cells (density of 100,000 cells/mL) were exposed for 24 h (37 °C, 97 % humidity, 5 % CO_2_) with a dilution series of the extracts, a solvent control (0.1 % DMSO), a negative control (cell medium: DMEM/F-12 without phenol red, supplemented with stripped foetal bovine serum and minimal essential medium) and a positive control (sodium lauryl sulphate; 150 μg/mL). Subsequently, the exposure medium was discarded, neutral red solution (33.33 μg/mL) was added and the cells were incubated at the above-mentioned conditions. After 2 h, the cells were washed twice with phosphate-buffered saline, and an acetic acid/ethanol-solution was added and shaken for 20 min. Finally, the NR was measured by fluorescence reading (excitation: 530 nm, emission: 645 nm), using the Tecan Spark^Ⓡ^ (Tecan Trading AG, Männedorf, Switzerland). The raw data from each replicate along with the respective pipetting scheme is provided in the file “EBM_raw_data.xlsx” in the sheet “NR_raw”. The cytotoxicity was calculated from blank corrected fluorescence readings in relation to the solvent control and is provided in the file “NR.csv”. Moreover, the 20 % inhibitory concentration (IC_20_) specified in the file “EBM.csv” was estimated in Prism 9 (GraphPad Software, Boston, MA, USA) with a two-parametric non-linear logistic regression.

#### 4.3.2 Chemically activated luciferase gene eXpression assay

The CALUX^Ⓡ^ test system, a set of mechanism-specific *in vitro* reporter gene assays, was used to evaluate the potential for endocrine disruption and oxidative stress-related effects. These assays are based on the human osteosarcoma cell line U2-OS, transfected with a firefly luciferase gene and coupled to a responsive element of interest such as the oestrogen receptor alpha (ERα), the androgen receptor (AR), the glucocorticoid receptor (GR), the progesterone receptor (PR) and the transcription factor Nrf2 (Nrf2). Activation of the responsive element leads to the production of associated proteins as well as luciferase, which induces light emission when luciferin is added as a substrate, allowing the assay to determine estrogenic, androgenic, glucocorticoid and progesterone effects and the potential to induce oxidative stress by luminescence measurement.

The agonistic (ERα-, AR-, GR- and PR-CALUX^Ⓡ^), antagonistic (anti-ERα-, anti-AR-CALUX^Ⓡ^) and Nrf2-CALUX^Ⓡ^ assays were performed as described in van der Linde et al. [15,16]. In short, the corresponding cells (density of 100,000 cells/mL) were seeded in 96-well plates and incubated for 24 h (37 °C, 97 % humidity, 5 % CO_2_) in assay medium (DMEM/F-12 without phenol red, supplemented with stripped foetal bovine serum and minimal essential medium). The medium was then removed and replaced with exposure medium containing a non-cytotoxic dilution series of the samples and a dilution series of the respective reference substances (17β-oestradiol for ERα, dihydrotestosterone for AR, dexamethasone for GR, medroxyprogesterone acetate for PR, tamoxifen for anti-ERα, flutamide for anti-AR and curcumin for Nrf2). For antagonistic assays, the exposure medium was additionally spiked with the reference compound of the agonistic assay at a concentration corresponding to the EC_50_. After incubation under the above-mentioned conditions for a further 24 h, the exposure medium was removed, the cells were lysed using lysis buffer (25 mM TRIS, 2 mM 1,4-dithiothreitol, 2 mM 1,2-diaminocyclohexanetetraacetic acid disodium salt, 10 % glycerol, 1 % Triton X^Ⓡ-^100), luciferin substrate solution (20 mM tricine, 1.07 mM (MgCO_3_)_4_Mg(OH)_2_·5H_2_O, 2.67 mM MgSO_4_·7H_2_O, 0.1 mM EDTA, 1.5 mM 1,4-dithiothreitol, 539 μM d-Luciferin, 5.49 mM ATP) was added and the resulting luminescence was quantified using a Tecan Spark^Ⓡ^ multimode reader (Tecan Trading AG, Männedorf, Switzerland). The determination of bioanalytical equivalents and limits of quantification from luminescence readings were performed in Excel (Microsoft Corporation, Redmond, Washington, USA) using macro-enabled templates provided by BioDetection Systems BV, following the calculations described in van der Linde et al. [15,16] and references therein. Briefly, the bioanalytical equivalents were calculated, based on the relative effect potency, comparing the effect-concentration relationship of the standard with that of the samples. The limits of quantification were determined based on replicated measurements of the solvent blank and calculated from the mean relative luminescence units and 10 times the standard deviation interpolated to the calibration curve of the respective standard dilution series. The validity of each replicate was evaluated based on the goodness of fit of the respective standard curve, the EC_50_ of the respective standard and the induction factor between the highest and lowest luminescence reading of the standard curve. The raw data for each bioassay and each replicate along with the respective pipetting scheme can be found in the online repository [1] in the file “EBM_raw_data.xlsx”. The processed data is provided in the file “EBM.csv”.

#### 4.3.3 Fish embryo acute toxicity test

Fertilised eggs of zebrafish (*Danio rerio*) were obtained for testing by mass spawning from the in-house zebrafish facility and collected within 2 h of spawning onset. Adult zebrafish (6–18 months) of a wild-type strain were used for rearing and groups of approximately 160 fish per tank (160 L) were reared in a flow-through system. Water quality was maintained by biofiltration followed by UV sterilisation in accordance with OECD Guideline 236. The water quality was monitored with an online multiprobe for temperature, pH and conductivity (Senect GmbH & Co. KG, Landau, Germany) as well as biweekly measurements of oxygen, water hardness, nitrite, nitrate and ammonia using commercial quick tests (JBL GmbH & Co. KG, Neuhofen, Germany and sera GmbH, Heinsberg, Germany). In addition, a weekly water exchange of approximately 40 % was achieved by automated addition of reverse osmosis-purified tap water reconstituted with Red Sea salt (Red Sea Deutschland, Düsseldorf, Germany) and sodium bicarbonate (food grade). The water temperature was maintained at 26 ± 2 °C and the lighting was adjusted to a light:dark rhythm of 14:10 h. The fish were fed daily with SDS small granular fish food (SDS, Witham, England) and live brine shrimp nauplii *Artemia sp*. (Great Salt Lake Artemia cooperative through ZebCare, Nederweert, The Netherlands).

In accordance with OECD Guideline 236 [17], the prolonged fish embryo acute toxicity test (FET) up to 120 h post fertilisation (hpf) was performed in three independent replicates to evaluate the teratogenic potential of individual river water extracts. Briefly, 20 embryos per concentration and replicate were statically exposed to a dilution series of the extracts shortly after fertilisation in 96-well plates and incubated at 26 ± 1 °C with a 14:10 h light:dark cycle. Lethal and sublethal effects (Table 3) as well as successful hatching of embryos were recorded every 24 h. All experiments were terminated by euthanasia of larvae shortly before 120 hpf, as zebrafish larvae younger than 120 hpf are not protected animal stages according to EU Directive 2010/63/EU [18].

**Table 3:** Lethal and sublethal criteria scored during the fish embryo toxicity test.

Lethal criteria	Sublethal criteria
Coagulation of the eggs	Spinal deformations
No heartbeat	Reduced heartbeat / reduced blood circulation
Lack of somites	Reduced / no pigmentation
Non-detachment of the tail	Oedema

From the recorded effects matching the lethal criteria (Table 3), the mortality percentages were calculated for each replicate relative to the total number of embryos tested. The same was done for all combined effects (i.e. meeting lethal and/or sublethal criteria) to calculate the percentage effects. Additionally, hatching success was determined from the total number of hatched larvae in relation to the total number of larvae tested. Following the validity criteria in OECD Guideline 236, the validity of each replicate was checked and only valid replicates were used for further analysis. The data for the timepoint 119 hpf is summarised in the file “FET.csv”. Furthermore, using a two-parametric non-linear logistic regression in Prism 9 (GraphPad Software, Boston, MA, USA), lethal and effect concentrations (LCs and ECs) for 5, 10, 20 and 50 % of the tested population were computed and are provided in the file “EBM.csv”.

## Limitations

Due to insufficient sample volumes, only two valid replicates could be completed for the Nrf2-CALUX^Ⓡ^ and one valid replicate each for the GR and PR-CALUX^Ⓡ^ assays. However, these limitations can be considered relatively minor as the data obtained for the GR- and PR-assays showed no detectable effects and can therefore be considered as a first no-effect screening. For the Nrf2 data, two replicates can be considered a sufficient basis for effect determination, although a third replicate would be favourable. Furthermore, no Nrf2 data were obtained for the trip blank sample, so an oxidative stress-related effect of the blank cannot be excluded. As the chemical analysis was conducted on a target list, the data might not sufficiently represent all possible compounds that exert an adverse effect on organisms inhabiting the aquatic environment. Furthermore, only a limited list of water parameters was determined. These limitations are in principle important to consider when evaluating the dataset.

## Funding

This work received funding from the RobustNature Cluster of Excellence Initiative provided by the Goethe University Frankfurt, Germany. The Q Exactive Plus LC-HRMS used at UFZ is part of the major infrastructure initiative CITEPro (Chemicals in the Terrestrial Environment Profiler) funded by the Helmholtz Association.

## Ethics Statement

We hereby confirm that we have read and follow the ethical requirements for publication in Data in Brief and confirm that the current work does not involve human subjects, animal experiments, or any data collected from social media platforms.

## CRediT authorship contribution statement

**Fabian G. Weichert:** Conceptualization, Data curation, Formal analysis, Investigation, Methodology, Validation, Visualization, Writing – original draft, Writing – review & editing, Funding acquisition. **Werner Brack:** Resources, Writing – review & editing. **Mario Brauns:** Conceptualization, Writing – review & editing. **Patrick Fink:** Conceptualization, Writing – review & editing. **Sarah Johann:** Conceptualization, Methodology, Formal analysis, Writing – review & editing, Funding acquisition. **Martin Krauss:** Methodology, Validation, Investigation, Writing – review & editing. **Henner Hollert:** Resources, Writing – review & editing, Funding acquisition.

## Data Availability

Chemical analysis dataset for contaminants of emerging concern and bioanalytical data for samples from a low mountain stream in Central Germany (Original data).

## References

[1] F.G. Weichert, W. Brack, M. Brauns, P. Fink, S. Johann, M. Krauss, H. Hollert, Chemical analysis dataset for contaminants of emerging concern and bioanalytical data for samples from a low mountain stream in Central Germany, (2024). 10.5281/zenodo.10892038. PMC11127143 38799712

[2] Altenburger R., Brack W., Burgess R.M., Busch W., Escher B.I., Focks A., Hewitt L.M., Jacobsen B.N., de Alda M.L., Ait-Aissa S., Backhaus T., Ginebreda A., Hilscherova K., Hollender J., Hollert H., Neale P.A., Schulze T., Schymanski E.L., Teodorovic I., Tindall A.J., Umbuzeiro G.D., Vrana B., Zonja B., Krauss M. (2019). Future water quality monitoring: improving the balance between exposure and toxicity assessments of real-world pollutant mixtures. Environ. Sci. Eur..

[3] Wolfram J., Stehle S., Bub S., Petschick L.L., Schulz R. (2021). Water quality and ecological risks in European surface waters – monitoring improves while water quality decreases. Environ. Int..

[4] Sylvester F., Weichert F.G., Lozano V.L., Groh K.J., Bálint M., Baumann L., Bässler C., Brack W., Brandl B., Curtius J., Dierkes P., Döll P., Ebersberger I., Fragkostefanakis S., Helfrich E.J.N., Hickler T., Johann S., Jourdan J., Klimpel S., Kminek H., Liquin F., Möllendorf D., Mueller T., Oehlmann J., Ottermanns R., Pauls S.U., Piepenbring M., Pfefferle J., Schenk G.J., Scheepens J.F., Scheringer M., Schiwy S., Schlottmann A., Schneider F., Schulte L.M., Schulze-Sylvester M., Stelzer E., Strobl F., Sundermann A., Tockner K., Tröger T., Vilcinskas A., Völker C., Winkelmann R., Hollert H. (2023). Better integration of chemical pollution research will further our understanding of biodiversity loss. Nat. Ecol. Evol..

[5] Wang Z., Walker G.W., Muir D.C.G., Nagatani-Yoshida K. (2020). Toward a global understanding of chemical pollution: a first comprehensive analysis of national and regional chemical inventories. Environ. Sci. Technol..

[6] Malaj E., von der Ohe P.C., Grote M., Kühne R., Mondy C.P., Usseglio-Polatera P., Brack W., Schäfer R.B. (2014). Organic chemicals jeopardize the health of freshwater ecosystems on the continental scale. PNAS.

[7] Brack W., Aissa S.A., Backhaus T., Dulio V., Escher B.I., Faust M., Hilscherova K., Hollender J., Hollert H., Müller C., Munthe J., Posthuma L., Seiler T.-B., Slobodnik J., Teodorovic I., Tindall A.J., de Aragão Umbuzeiro G., Zhang X., Altenburger R. (2019). Effect-based methods are key. The European Collaborative Project SOLUTIONS recommends integrating effect-based methods for diagnosis and monitoring of water quality. Environ. Sci. Europe.

[8] Backhaus T. (2023). Commentary on the EU commission's proposal for amending the water framework directive, the groundwater directive, and the directive on environmental quality standards. Environ. Sci. Eur..

[9] Weitere M., Altenburger R., Anlanger C., Baborowski M., Bärlund I., Beckers L.-M., Borchardt D., Brack W., Brase L., Busch W., Chatzinotas A., Deutschmann B., Eligehausen J., Frank K., Graeber D., Griebler C., Hagemann J., Herzsprung P., Hollert H., Inostroza P.A., Jäger C.G., Kallies R., Kamjunke N., Karrasch B., Kaschuba S., Kaus A., Klauer B., Knöller K., Koschorreck M., Krauss M., Kunz J.V., Kurz M.J., Liess M., Mages M., Müller C., Muschket M., Musolff A., Norf H., Pöhlein F., Reiber L., Risse-Buhl U., Schramm K.-W., Schmitt-Jansen M., Schmitz M., Strachauer U., von Tümpling W., Weber N., Wild R., Wolf C., Brauns M. (2021). Disentangling multiple chemical and non-chemical stressors in a lotic ecosystem using a longitudinal approach. Sci. Total Environ..

[10] Schulze T., Ahel M., Ahlheim J., Ait-Aissa S., Brion F., Di Paolo C., Froment J., Hidasi A.O., Hollender J., Hollert H., Hu M., Klolss A., Koprivica S., Krauss M., Muz M., Oswald P., Petre M., Schollee J.E., Seiler T.B., Shao Y., Slobodnik J., Sonavane M., Suter M.J.F., Tollefsen K.E., Tousova Z., Walz K.H., Brack W. (2017). Assessment of a novel device for onsite integrative large-volume solid phase extraction of water samples to enable a comprehensive chemical and effect-based analysis. Sci. Total Environ..

[11] Finckh S., Beckers L.-M., Busch W., Carmona E., Dulio V., Kramer L., Krauss M., Posthuma L., Schulze T., Slootweg J., Von der Ohe P.C., Brack W. (2022). A risk based assessment approach for chemical mixtures from wastewater treatment plant effluents. Environ. Int..

[12] T. Schulze, E. Müller, M. Krauss, MZquant - high-throughput target screening, (2021). https://git.ufz.de/wana_public/mzquant.

[13] U.S.E.P.A. US EPA, Definition and Procedure for the Determination of the Method Detection Limit, Revision 2, United States Environmental Protection Agency, 2016. https://www.epa.gov/sites/default/files/2016-12/documents/mdl-procedure_rev2_12-13-2016.pdf.

[14] Repetto G., del Peso A., Zurita J.L. (2008). Neutral red uptake assay for the estimation of cell viability/cytotoxicity. Nat. Protoc..

[15] van der Linden S.C., Heringa M.B., Man H.-Y., Sonneveld E., Puijker L.M., Brouwer A., van der Burg B. (2008). Detection of multiple hormonal activities in wastewater effluents and surface water, using a panel of steroid receptor CALUX bioassays. Environ. Sci. Technol..

[16] van der Linden S.C., von Bergh A.R.M., van Vught-Lussenburg B.M.A., Jonker L.R.A., Teunis M., Krul C.A.M., van der Burg B. (2014). Development of a panel of high-throughput reporter-gene assays to detect genotoxicity and oxidative stress. Mutat. Res./Genet. Toxicol. Environ. Mutagenesis.

[17] OECD, (2013).

[18] Strähle U., Scholz S., Geisler R., Greiner P., Hollert H., Rastegar S., Schumacher A., Selderslaghs I., Weiss C., Witters H., Braunbeck T. (2012). Zebrafish embryos as an alternative to animal experiments—a commentary on the definition of the onset of protected life stages in animal welfare regulations. Reprod. Toxicol..

